# Dissecting the Lymphatic System to Predict Melanoma Metastasis

**DOI:** 10.3389/fonc.2020.576190

**Published:** 2020-11-27

**Authors:** Rishi Suresh, Arturas Ziemys, Ashley M. Holder

**Affiliations:** ^1^ Texas A&M College of Medicine, Bryan, TX, United States; ^2^ Department of Nanomedicine, Houston Methodist Research Institute, Houston, TX, United States; ^3^ Department of Surgery, Division of Surgical Oncology, University of Alabama at Birmingham, Birmingham, AL, United States

**Keywords:** lymphatics, melanoma, metastasis, lymphangiogenesis, transport

## Abstract

Melanoma is the most lethal form of skin cancer in the United States. Current American Joint Committee on Cancer (AJCC) staging uses Breslow depth and ulceration as the two primary tumor factors that predict metastatic risk in cutaneous melanoma. Early disease stages are generally associated with high survival rates. However, in some cases, patients with thin melanomas develop advanced disease, suggesting other factors may contribute to the metastatic potential of an individual patient’s melanoma. This review focuses on the role of the lymphatic system in the metastasis of cutaneous melanoma, from recent discoveries in mechanisms of lymphangiogenesis to elements of the lymphatic system that ultimately may aid clinicians in determining which patients are at highest risk. Ultimately, this review highlights the need to integrate pathological, morphological, and molecular characteristics of lymphatics into a “biomarker” for metastatic potential.

## Introduction

Melanoma is responsible for the majority of skin cancer related deaths in the United States. Despite an increased incidence in the United States, melanoma mortality has decreased significantly in the past few years (1). However, metastatic melanoma still carries a poor prognosis. The American Joint Committee on Cancer (AJCC) staging system, 8th edition, has identified Breslow depth and ulceration as important predictive factors of survival in patients with melanoma (2). When detected at an early stage, melanoma can be treated with wide local excision and staged with sentinel lymph node biopsy. However, more advanced disease requires a multidisciplinary approach that often includes systemic therapy—from targeted treatments (BRAFi) to checkpoint inhibitors (3). Nonetheless, up to 15% of patients who have thin melanomas ultimately develop metastatic disease (4, 5). The best way to identify these high-risk patients, manage their nodal basin, and improve their survival remains controversial (5). Therefore, further study is needed to improve risk stratification and staging of melanoma.

Melanoma preferentially metastasizes to lymph nodes, leading to hypotheses that it spreads through the lymphatic vasculature (4). However, the exact mechanisms of lymphatic invasion and metastasis are not well-defined. Recently, experimental models have been developed that explore the role of growth factors—such as VEGF-C—in lymphangiogenesis and eventual melanoma metastasis (6). Furthermore, several studies have closely analyzed the alterations that occur in the lymphatic system in response to melanoma, including changes in vessel size, density, and transport kinetics (7–9). Together, these studies have suggested that the lymphatic system likely has an essential function in melanoma metastasis. This review will assess how the lymphatic system may contribute to metastasis of cutaneous melanoma, specifically focusing on factors that predict metastatic potential and can be integrated into a lymphatic “biomarker.”

## Lymphatic Density, Lymphatic Invasion, and Melanoma Metastasis

Given that melanomas often spread to lymph nodes, others have hypothesized that melanomas that are likely to metastasize would demonstrate increased lymphatic vessel density (LVD). Early attempts to study lymphatic vasculature in melanoma were limited by the challenge of distinguishing blood vessels from lymphatics, leading some initial studies to conclude that lymphatic density was unchanged in metastatic melanoma (10). Once antibodies specific for lymphatic vasculature in the skin, such as LYVE-1, were developed, investigating lymphatics was possible (7). To test the hypothesis that tumors that metastasize to the lymph nodes would show increased lymphatic density, Shields et al. compared lymphatic density in melanoma to: 1) normal dermis, 2) basal cell carcinoma (BCC), and 3) Merkel cell carcinoma (MCC). Both intratumoral and epitumoral lymphatic density were found to be substantially increased in melanoma relative to BCC or MCC, suggesting that lymphatic density is increased in tumors that preferentially metastasize to lymph nodes. Furthermore, increased lymphatic density was found to be associated with melanomas that were more likely to metastasize than those that were not. Finally, melanomas included in their study that had both vascular and lymphatic invasion were frequently metastatic. These results point to the possible value of utilizing both lymphatic vessel density and lymphovascular invasion as important predictive features in assessing metastatic potential.

Together, these observations were incorporated into the Shields index, a predictive metric based on lymphatic invasion, lymphatic density, and Breslow thickness to the metastatic potential of an individual melanoma. While the initial Shields et al. study had a relatively small sample size of 21 melanomas, several subsequent studies have reported that increased lymphatic density leads to a poor prognostic outcome (4, 7, 11, 12). Both Emmett et al. and Spiric et al. attempted to use the Shields index to predict whether a melanoma was likely to metastasize (**Table 1**). In a retrospective review of 102 melanomas, Emmett et al. found that the Shields index was the best technique for discriminating between metastatic and non-metastatic melanomas, followed by lymphatic vessel density alone, AJCC staging, and Breslow thickness (4). Similarly, Spiric et al. reviewed 100 melanoma specimens and found that the Shields index performed better than either melanoma thickness or AJCC staging at predicting metastatic potential of melanoma (7, 11).

**Table 1:** The Shields index to predict metastatic potential of cutaneous melanoma.

Authors	Sample size	Design	Epitumoral lymphatic vessel density method	Outcome
Shields et al. (7)	21	Retrospective	Complete • All lymphatics counted • x40 objective • 350 µm from tumor edge	Index predicted metastasis more effectively than thickness alone
Emmett et al. (4)	102	Retrospective	Complete and hotspot • x100 objective • Three areas of subjectively high density were counted and averaged	Hotspot faster but comparable to complete method Shields index (81% specific, 82% sensitive) predicted metastasis more effectively than lymphatic vessel density and AJCC staging
Spiric et al. (11)	100	Retrospective	Hotspot	Shields index (75% specific, 81.3% sensitive), performed better at predicting metastasis than thickness and AJCC staging

Nonetheless, the role of lymphovascular invasion as a unique predictive value in determining metastasis is unclear. A review of 1,029 melanomas from the Melbourne Melanoma Project found lymphatic invasion to be a significant predictor of recurrence (13). However, other studies concluded that lymphatic invasion is not a significantly unique predictor of melanoma metastasis (14–19). More recent national cancer database analyses demonstrated that lymphovasacular invasion by histopathological analysis is an independent predictor of sentinel lymph node metastasis in patients with T2 (20) but not T1 (17) melanoma, while a European multi-institutional study suggested that lymphovascular invasion was an independent predictor of sentinel lymph node metastasis in patients with T1b melanoma (21). The discrepancies in these findings reveal the complexity of dissecting the role of lymphovascular invasion in metastasis. Notably, several studies that found no association between lymphatic invasion and metastasis had large sample sizes, further contributing to this controversy (**Table 2**).

**Table 2:** Studies concluding that lymphatic invasion does not predict metastasis.

Authors	Sample size	Staining technique	Outcome
Pettit et al. (16)	27 10 specimens with LI	D2-40/S-100 dual Immunohistochemistry	Lymphatic Invasion (LI) not associated with SLN metastasis
Egger et al. (17)	6894 T1b Melanoma specimens 107 specimens with LI	n/a	LI not a significant predictor of metastasis in T1b melanoma
Storr et al. (18)	202 specimens of thickness ≥ 0.75mm 27 specimens with LI	D2-40/CD34	No association with clinical outcome (relapse free or overall survival)
Rose et al. (19)	246 specimens (18% with LI)	D2-40/CD34	LI not significant predictor of SLN status

LI, lymphatic invasion.

These studies suggest that lymphatic vessel density (LVD) and the Shields index are valuable predictive tools. One possible reason that LVD has not been adopted as a predictive factor in clinical practice is the time-intensive nature of the procedure. In their comparison of different methods of identifying LVD, Emmett et al. compared the traditional Shields method to the “hot spot method.” In the traditional Shields method, every lymphatic within 350 µm of the tumor edge is counted. The hot spot method requires that only three areas of subjective high lymphatic density are counted and then averaged. When compared, the time to complete the traditional Shields method was 19 min per slide, while the hot spot analysis was only 5.5 min (7). Despite being about four times faster, the hot spot method did not yield a Shields index that was significantly different than the traditional method. However, even the more cumbersome method of calculating lymphatic vessel density has the potential to provide important prognostic information for melanoma patients.

## Mechanisms of Lymphangiogenesis

The mechanism of tumor spread from the primary to the sentinel lymph node has been investigated in both animal models and patients (**Table 3**). Studies have demonstrated that tumor-draining lymph nodes enlarge prior to clinical evidence of metastasis (8). However, the specific alterations and mechanisms that lead to these changes are poorly understood.

**Table 3:** Mechanistic studies evaluating melanoma metastasis.

Authors	Model	Key findings
Hoshida et al. (22)	B16F10 melanoma with C57BL/6 mice	VEGF-C overexpressing cell lines increased tumor cell delivery and flow rate
Qian et al. (8)	Human NPC cell line in BALB/c mice	Tumor-draining lymph nodes can enlarge prior to evidence of metastasis
Harrell et al. (23)	B16F10 melanoma with C57BL/6 mice	Increase in size of lymphatic sinuses and flow in draining lymph node of tumor
Fankhauser et al. (6)	B16F10 melanoma model	Blocking VEGF Receptor-3 inhibited lymphangiogenesis and immunosuppressive cell infiltration
Lund et al. (24)	B16F10 melanoma with C57BL/6 mice	VEGF-C associated with infiltration of Treg into primary tumor; tumors with VEGF-C expression suppressed naïve T cell activation
Lane et al. (25)	C57BL/6J mice with B16F10.OVA, MC38, YUMM1.7, YUMMER1.7 cells	Lymphatic endothelium plays a critical role in creating an immunosuppressive environment permitting tumor growth
Commerford et al. (26)	B16F10 melanoma with BALB/c and C57BL/6 mice	Adhesion genes such as Jam3 or integrin aIIb differentially regulated in tumor draining lymph nodes

VEGF-C has been studied extensively for its role in melanoma growth and metastasis. Hoshida et al. evaluated lymphatic drainage in C57BL/6 mice injected with the B16F10 murine melanoma cell line using intravital microscopy. To confirm prior reports that VEGF-C is involved in lymphangiogenesis, their team developed VEGF-C overexpressing cell lines and noticed that tumor cell delivery and lymph flow rate increased in the draining lymph nodes in models of this cell line (22). This finding was largely confirmed by Harrell et al. who injected B16F10 into the footpad of C57BL/6 mice and noted a dramatic increase in the size of lymphatic sinuses and flow in the draining lymph node (23). Interestingly, the study did not identify any alterations in the lymphatics immediately adjacent to the tumor. Their result is inconsistent with findings in human patients but may be related to the creation of the murine model compared to primary melanomas from patients (4, 7, 11). Finally, blocking the VEGF receptor-3 (a key receptor of VEGF-C) in a murine B16F10 melanoma model inhibited lymphangiogenesis and immunosuppressive cell infiltration, further suggesting that VEGF-C plays an important role in the growth of lymphatic vessels in response to tumor (6). Furthermore, in a similar murine model, the expression of VEGF-C was associated with infiltration of immunosuppressive cells, including regulatory T cells into the primary tumor (24). Tumors with VEGF-C expression were shown to suppress naïve T cell activation in the draining lymph node, even more strongly than preexisting vaccine-induced immunity (24).

It is important to note that several of the aforementioned studies employ a syngeneic B16F10 melanoma model, which have been previously shown to have differences in vascularity and other structural irregularities compared to human melanoma (27). In addition, differences in the immunogenicity and genetic background (lack of BRAF mutation) have called into question the applicability of this murine melanoma model (28, 29). Therefore, the biology revealed from research using these models may not translate to patients.

## Lymphatic Vessels and the Tumor Microenvironment

Given the role of lymphatic vessels in regulating immunologic tolerance in normal environments, Lane et al. hypothesized that they may regulate tumor environments (25). Their results from murine models found that non-hematopoietic PD-L1 is expressed in lymphatic endothelial cells (LEC), limiting CD8+ T cell accumulation. They further established that IFNγ released by activated CD8+ T cells can induce PD-L1 expression in LECs and that loss of IFNγ receptor leads to increased T cell accumulation. These results suggest that the lymphatic endothelium has a critical function in developing an immunosuppressive environment that permits tumor growth. Implantation of B16F10 into B lymphocyte-deficient mice demonstrated that lymphatic network size and flow did not increase in the draining lymph nodes (23). Furthermore, primary tumors implanted into the footpad of mice were noted to attract myeloid cells and macrophages, but the tumor draining lymph nodes would accumulate T and B lymphocytes, suggesting that B lymphocytes are critical for the changes observed in distant lymph nodes (23). Together, these results suggest that immunosuppressive effects of melanoma are complex and mediated by B lymphocytes, lymphatic endothelium, and growth factors such as VEGF-C.

From these studies, targeting VEGF-C was considered to be a promising approach for decreasing metastasis in melanoma. However, the AVAST-M trial using adjuvant bevacizumab for melanoma patients failed to identify a significant difference in survival at 5 years (30, 31). Findings from studies, including Fankhauser et al., suggest that the effect of immunotherapy is potentiated in tumors that cause increased lymphangiogenesis. In a murine model, adoptively transferred *ex vivo* activated CD8+ T cells were able to respond to tumors in which VEGF-C was expressed compared to tumors where VEGFR-3 was blocked (6). To extrapolate their findings beyond mice, the sera of human metastatic melanoma patients were tested for VEGF-C. Consistent with their findings in murine models, higher VEGF-C concentrations correlated with response to immunotherapy and progression free survival.

Future studies may identify additional therapeutic targets of the lymphatic system in melanoma metastasis. Commerford et al. performed RNA sequencing of the LECs in the tumor draining lymph node of mice injected with B16F10 melanoma and compared it to normal LECs (26). Cell-cell and cell-matrix adhesion genes such as Jam3 or integrin αIIb were differentially regulated in the tumor-draining lymph node, suggesting that LECs in tumor-draining lymph nodes are altered at a transcriptional level. These results could identify potential future targets to prevent lymphangiogenesis in metastatic melanoma. Additional research needs to be performed to translate these mechanistic insights in routine clinical practice. Generally, the biomarkers associated with melanoma metastasis are diluted in a routine blood draw, limiting their use (32). Broggi et al. described use of postoperative lymphatic exudate and plasma in stage III melanoma patients as a way to collect biomarkers including factors not only associated with melanoma (LDH, S100B, S100A8) but also linked to metastatic potential (CSF-1, galectin-3, MMP2- MM-9). While this method could be used in patients with advanced disease, the difficulty of accessing lymphatic exudate limits its use in earlier stage melanomas in which extensive lymph node dissection is not typically performed.

Furthermore, recent research has shown that the tumor microenvironment could potentially be affected following surgical alterations. Following inoculation of B16F10 melanoma cells into BALB/c mice, Nakamura et al. performed bilateral inguinal lymph node resection or a U-incision and noted that tumor growth was significantly increased in mice with surgical damage (33). Upon further histologic analysis of the tumor, they noted that both the total number of CD4+ and CD8+ T cells and apoptotic cells were significantly reduced in the mice that underwent surgical intervention. These observations were also seen when using an immunogenic tumor cell line (MC38). Therefore, adaptive immunity mechanisms may be impaired by the disruption of lymphatic vasculature following surgery, due to impaired transit of tumor antigens through lymphatic vasculature to regional lymph nodes and subsequent expansion of tumor-specific T-cells.

## Lymphatic Transport Kinetics

The kinetics of lymphatic transport have also been studied for prognostic applications in melanoma (**Table 4**). In a prospective trial of 276 patients, technetium-99m based lymphoscintigraphy was used to determine whether a patient had fast (less than 20 min) or slow (greater than 20 min) lymphatic transit (34). In this small feasibility study, all patients with slow drainage were found to be disease-free at 2 year follow-up. Later studies attempted to determine whether this scintigraphic appearance time (SAT) would be a reliable factor to distinguish melanomas based on their metastatic potential. Cammilleri et al. performed lymphoscintigraphy on 88 subjects with limb and trunk melanomas and retrospectively was able to determine that an SAT greater than 30 min correlated with a negative predictive value of 100% for the sentinel lymph node (35). These early studies suggested that a retrospective distinction could be made between SLN positive and SLN negative patients based on SAT time. However, when Mahieu-Renard et al. applied a SAT time of 30 min in a prospective cohort of 150 patients, the study yielded a negative predictive value of only 84.6% (65.1–95.6%) (36). It is slightly surprising that a lymph node containing tumor might have faster drainage, given that tumor infiltration of lymph nodes would theoretically cause an obstruction of flow (36), but likely the flow is unobstructed until large amounts of tumor are present. However, as shown previously, metastatic melanoma has been shown to significantly increase peritumoral lymphatic density (7) and increase the size of tumor draining lymph vasculature (23), factors that would likely contribute to increased transport flow capacity. Furthermore, metastatic melanoma is likely to result in an increase in activated macrophages (23), leading to increased scintigraphic uptake.

**Table 4:** Studies evaluating lymphatic transport kinetics as predictive of sentinel lymph node metastasis.

Authors	Samplesize	Location of melanoma	Melanoma features	Type of melanoma^a^	Design	Colloid	Time cutoffs^b^	Variable^c^	Results^d^
Maza et al. (34)	276	Trunk, lower limbs, upper limbs, head/neck	pT1-T4	SS, NM, LM, AL	Prospective	Tc-99 nanocolloid	20 min	SAT	No SLN metastasis in slow drainage group
Cammilleri et al. (35)	88	Trunk, upper limb, lower limb	Stage I and II	n/a	Prospective	Tc-99m colloidal rhenium sulfide	30 min	SAT	No SLN metastasis in slow drainage group
Mahieu-Renard et al. (36)	Retro: 194 Prosp: 150	Limbs, trunk, hands/feet, head/neck	Breslow: ≤1mm->4mm	SS, NM, AL, LM	Retrospective, prospective	Tc-99m colloidal rhenium sulfide	30 min	SAT	Slow lymphatic drainage had a negative predictive value of 84.6%
Fujiwara et al. (9)	11	Trunk	n/a	SS	Retrospective	Tc-99m phytate	30 min	SST, LTR	All SLNs with <1.8cm/min LTR were non-metastatic
Toubert et al. (37)	160	Upper limb, lower limb, trunk, head/neck	Breslow >1mm	n/a	Prospective	Tc-99m colloidal rhenium sulfide	30 min	SAT	No significant difference based on speed of drainage

^a^Superficial spreading (SS), lentigo maligna (LM), acral lentiginous (AL), nodular melanoma (NL).

^b^Lymphatic transport rate (LTR).

^c^Scintigraphic appearance time (SAT), scintigraphic saturation time (SST), lymphatic transport rate (LTR).

^d^Sentinel lymph node (SLN).

More recently, Fujiwara et al. described the use of the area extraction method to evaluate lymphatic kinetics in patients with truncal melanoma (9). The method utilizes technetium-99m phytate to perform dynamic lymphoscintigraphy and uses a gamma camera to acquire images and develop time activity curves (plotting tracer counts against time). Using this data, the researchers were able to identify a plateau, which they established as the scintigraphic saturation time (SST). Compared to the prior efforts of estimating SAT which were primarily based on researcher visualization, the SST represents a more reproducible method since it does not depend on the researcher’s visualization. To determine the lymphatic transit rate (LTR), the distance between the primary tumor and the SLN (using real-time fluorescence navigation with indocyanine green) was calculated and then divided by the SST. Together, the LTR and SST were found to be significant in determining the status of the sentinel lymph node in patients with melanoma (9).

While there is evidence to support the notion that SAT can possibly determine the likelihood of sentinel lymph node positivity, some studies have contrarily suggested that there is no significant difference in SLN metastasis and speed of lymphatic transport. For example, Toubert et al. found in a cohort of 160 patients that there was no difference in metastatic SLN based upon speed of drainage using dynamic acquisition and static imaging divided into fast (<20 min), intermediate (20–30 min) or slow (>30 min) lymphatic drainage (37). Lymphoscintigraphy and lymphatic transport are factors that are difficult to standardize, which could account for the differences in procedures. Lymphatic transport can be affected broadly by several factors including age (38), weight (39), musculature, changes in Starling forces, and body position (40). The size of the colloid also significantly affects the SAT. Both Camilleri et al. and Toubert et al. used the same colloid (99mTc-rhenium sulfide) and therefore utilized the same SAT in their respective studies. Maza et al. used a 99mTc-nanocolloid, accounting for the difference in SAT (34). Both of these colloids were smaller than the 99mTc-phytate employed by Fujiwara et al. (9).

Furthermore, the transit time of lymphatic fluid varies greatly based on region of the body. Specifically, lymphatic drainage rates are significantly lower in the head and neck relative to the extremities (41). Additionally, the use of lymphoscintigraphy in the head and neck or perineal region can be obscured by shine-through (9). Therefore, any protocol developed regarding the use of SAT as a predictive marker of SLN metastasis will require an individualized approach based on these factors to yield consistent, reproducible results. The area extraction method developed by Fujiwara et al. appears to be a standardized way of evaluating SST and LTR (9). However, their technique required multiple and frequent imaging. Nonetheless, techniques for assessing lymphatic transport efficiency will require standardization prior to integration into staging guidelines for melanoma, as differences in technique would likely result in discrepancies in the predictive value of these approaches.

## Discussion

The lymphatic system makes a critical contribution in melanoma metastasis. Recent studies have suggested that factors like VEGF-C and the lymphatic endothelium itself play an important role in altering the immune system to support melanoma metastasis (22, 25). While these studies often rely on B16F10 melanoma models that may not accurately replicate human biology, their findings may ultimately yield important mechanistic insights. Several studies have suggested that using lymphatic vessel density and lymphatic invasion in the Shields index may aid in determining the metastatic potential of melanoma (4, 7, 11),. Furthermore, utilizing lymphatic transport kinetics as a predictive factor appears to be a promising area of research (9, 35).

While these studies represent important steps toward understanding the role that the lymphatic system plays in the growth and metastasis of cutaneous melanoma, there are still several areas that require further study. The original Shields index was validated as a meaningful predictor of melanoma metastasis by the work of Emmett et al. and Spiric et al. (4, 11). However, all studies to date performed on the Shields index are retrospective in nature; thus, a prospective study is needed to establish the utility of the method. Furthermore, there are contradictory reports in the literature regarding features such as lymphatic invasion and their predictive potential, and future studies are needed to reconcile these differences. Additionally, while lymphatic transport kinetics have been shown to successfully identify metastatic melanomas, future research is needed to develop a reliable tool in clinical practice. The specific techniques utilized for analysis of lymphatic transport need to be refined to become more consistent and reproducible, and specialized protocols will need to be developed based on the type of colloid utilized and the affected body area. Finally, while experimental models have revealed significant findings in the role that melanoma plays in lymphangiogenesis, future study is required to translate these genetic and mechanistic insights into targeted therapies or biomarkers.

This review highlights several studies proposing the lymphatic system as a critical player in melanoma metastasis. Features such as lymphatic vessel density or lymphatic transport kinetics might eventually serve as adjuncts to current staging protocols to improve our ability to detect melanomas that are high-risk. Furthermore, future research on the lymphatic system and melanoma metastasis may aid in the development of biomarkers or novel targeted therapies. However, while the study of the lymphatic system may improve detection and management of melanomas that are likely to metastasize, there is the possibility of overtreating patients who otherwise could have been managed more conservatively. Future studies are necessary to develop a more accurate lymphatic “biomarker” that systematically integrates pathological, morphological, and molecular data to identify high-risk melanomas that are under-staged with current techniques.

## Author Contributions

RS, AZ, and AH jointly conceived the review. RS and AH performed the literature review and wrote the manuscript. AZ analyzed the quality of the transport analysis performed by studies included in the literature review. RS, AZ, and AH edited the manuscript at all stages. All authors contributed to the article and approved the submitted version.

## Conflict of Interest

The authors declare that the research was conducted in the absence of any commercial or financial relationships that could be construed as a potential conflict of interest.
